# The diaphragm-sparing effect of interscalene block with a low-volume of ropivacaine 0.1% vs. 0.5%: A double-blind, controlled, randomised trial

**DOI:** 10.1097/EA9.0000000000000108

**Published:** 2026-04-23

**Authors:** Anne-Laure Verbeke, Antoine Jeanneteau, Julie Bellet, Michael Genin, Cedric Cirenei, Arnaud Alluin, Benoit Tavernier

**Affiliations:** From the CHU Lille, Pôle d’anesthésie-réanimation, and University Lille, CHU Lille, ULR 2694, METRICS: Évaluation des Technologies de santé et des Pratiques médicales, Lille, France (ALV, AJ, JB, MG, CC, AA, BT)

## Abstract

**BACKGROUND:**

Despite improvements, the incidence of hemidiaphragmatic paralysis following interscalene brachial plexus block (ISB) is still significant at 20–40%.

**OBJECTIVES:**

We tested the hypothesis whereby the use of a low volume of ropivacaine 0.1% reduces the incidence of hemidiaphragmatic dysfunction (compared with the same volume of ropivacaine 0.5%), while providing sufficient analgesia following arthroscopic shoulder surgery.

**DESIGN:**

Randomised controlled trial.

**SETTING:**

A tertiary university hospital.

**PATIENTS:**

Sixty patients undergoing shoulder arthroscopy under general anaesthesia.

**INTERVENTION:**

Ultrasound-guided ISB with 7 ml of ropivacaine 0.1% or ropivacaine 0.5%.

**MAIN OUTCOME MEASURES:**

The primary outcome was the incidence of hemidiaphragmatic dysfunction (a decrease in diaphragm excursion >25% of baseline) 30 min after ISB. The secondary outcomes included hemidiaphragmatic paralysis (a decrease in diaphragm excursion >75% of baseline), spirometry variables and postoperative analgesia.

**RESULTS:**

Hemidiaphragmatic dysfunction occurred in 7/30 (23%) patients in the experimental group and in 29/30 (97%) patients in the control group, *P* < 0.001; relative risk (95% CI): 0.24 (0.13 to 0.46). Hemidiaphragmatic paralysis occurred in one patient in the experimental group and 25 of the patients in the control group (*P* < 0.001). The median [IQR range] decrease (%) in forced vital capacity was −17 [−23 to −5] in the experimental group and −29 [−42 to −6] in the control group; *P* = 0.039). The difference in the median duration of effective analgesia between the experimental and control groups was not significant (9.8 h and 15.5 h, respectively; *P* = 0.23; Log-rank test; mean (95%CI) difference in restricted mean survival time: -3.2 h (-6.4 to 0.2); (*P* = 0.061), but more patients required morphine postoperatively in the experimental group (10/30 vs. 3/30; *P* = 0.028).

**CONCLUSIONS:**

ISB with 7 ml of ropivacaine 0.1% is associated with a lower incidence of hemidiaphragmatic dysfunction, when compared with the same volume of ropivacaine 0.5%, but at the cost of potentially less effective postoperative analgesia.

**TRIAL REGISTRATION:**

https://clinicaltrials.gov/ct2/show/NCT04173364

**KEY POINTS**

- Interscalene block (ISB) with 7 ml of ropivacaine 0.1% was associated with a lower incidence of hemidiaphragmatic dysfunction than ISB with the same volume of ropivacaine 0.5%.
- ISB with 7 ml of ropivacaine 0.1% was associated with a lower incidence of hemidiaphragmatic paralysis than ISB with the same volume of ropivacaine 0.5%.
- Respiratory function (as assessed by spirometry) was less altered following an ISB with 7 ml of ropivacaine 0.1% as compared with the same volume of ropivacaine 0.5%.
- Postoperative analgesia obtained with ISB with 7 ml ropivacaine 0.1% after arthroscopic surgery appeared less effective than with ISB with the same volume of ropivacaine 0.5%.

## Introduction

Shoulder surgery is associated with significant postoperative pain. Interscalene block (ISB) of the brachial plexus is one of the most reliable regional analgesia techniques; it helps to decrease pain levels and opioid consumption, shorten the hospital stay, and increase patient satisfaction.^1,2^ However, ISB is associated with high incidences (up to 100%) of hemidiaphragmatic paralysis (usually defined as a reduction of more than 75% in diaphragmatic movement or paradoxical diaphragmatic movement during normal inspiration or deep inspiration), due to spreading of the local anaesthetic to the phrenic nerve or the C3–C5 nerve roots.^3^ This adverse event decreases the vital capacity and the forced expiratory volume in one second (FEV1)^3,4^ and may compromise ventilation and oxygenation^3,5^ – especially in patients with obesity or significant pulmonary disease.^3,6,7^ With the introduction of ultrasound guidance, the use of low volumes (up to 5 ml),^8^ low concentrations (up to 0.1%^9^) or extrafascial injections^10^ of local anaesthetic has been associated with a lower incidence of hemidiaphragm paralysis (20–40%) and the maintenance of adequate postoperative analgesia.^8–12^ To date, the combination of a low concentration of local anaesthetic and a low volume has not been evaluated in the context of ISB. We conducted a double-blind, controlled, randomised trial in patients undergoing arthroscopic shoulder surgery, in order to test the hypothesis whereby ISB with a low volume (7 ml) and a low concentration (0.1%) of ropivacaine would reduce the risk of hemidiaphragm dysfunction (relative to the same procedure with ropivacaine 0.5%) without altering levels of intra- and postoperative analgesia.

## Methods

This prospective, double-blind, controlled, randomised study was conducted at Lille University Hospital (Lille, France). The trial was approved by an independent ethics committee (CPP Est-II, Besançon, France; Chairperson: Prof Jean-Marc Chalopin; reference: 19/06/11/59953) on 8 July 2019, and registered at ClinicalTrials.gov (NCT04173364) on 21 November 2019. Written, informed consent was obtained from each patient prior to enrolment.

The main inclusion criteria were an American Society of Anesthesiologists (ASA) grade of I–III, age 18 or over, and scheduled elective shoulder arthroscopic surgery (rotator cuff repair, acromioclavicular resection, or biceps tenotomy) with ISB between November 2019 and September 2020 at Lille University Hospital. The main exclusion criteria were severe lung disease (such as stage III or IV chronic obstructive pulmonary disease, according to the Global Initiative for Chronic Obstructive Lung Disease rating system), coagulation disorders, pregnancy, and allergy to any of the study medications.

On the day of surgery, patients were allocated randomly 1 : 1 to the experimental group (ropivacaine 0.1%) or the control group (ropivacaine 0.5%), using a computer-generated randomisation table. Assignments were concealed in a sealed opaque envelope. A nurse not otherwise involved in the study or in patient management opened the envelope and prepared a 7 ml syringe containing the assigned drug, which was provided to the anaesthetist performing the ISB. The volume of 7 ml was selected because it was expected to ensure an analgesic effect in the control group at least equivalent to that reported by Riazi *et al.* for 5 ml of ropivacaine 0.5%.^8^ Thus, the patients, ISB operators, surgeons, nurses, and study evaluators were all blinded to the group allocation.

### Interscalene block procedure

**FIGURE 1:**
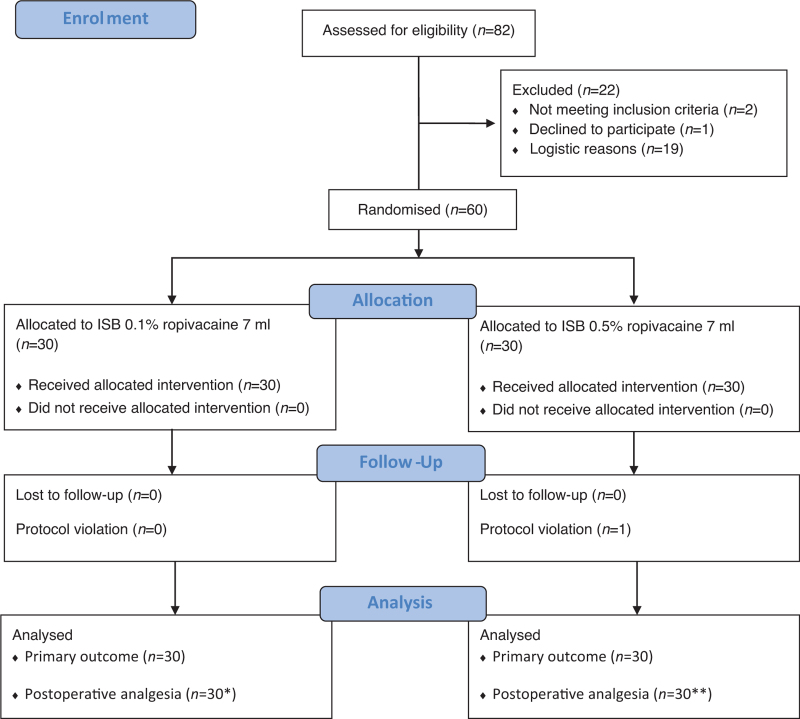
The study flow diagram, drawn in compliance with the Consolidated Standards of Reporting Trials (CONSORT).

^*^n = 29 in sensitivity analyses (see text). ^**^1 patient censored at time of protocol violation; *n* = 28 in sensitivity analyses (see text).

All ultrasound-guided ISBs were performed before surgery in a dedicated location in the holding area. The ISB was performed by an experienced anaesthetist with the patient in the supine position and the head turned to the nonoperating side, using a high-frequency (4–13 MHz) linear transducer (ML6–15 probe) connected to a LOGIC P9 ultrasound machine (GE Healthcare, Chicago, IL, USA) under sterile conditions. After identifying the cervical nerve roots and interscalene muscles, a 50-mm, 22-gauge insulated block needle was inserted using an out-of-plane approach, tangential to the middle scalene muscle, between the muscle and the C5 and C6 nerve roots (Figure 1, Supplemental Digital Content). Subsequently, 7 ml of ropivacaine (0.1% or 0.5%) were injected as a single slow intrafascial injection, while confirming appropriate spread of the local anaesthetic in contact with both nerve roots.

### Intraoperative and postoperative management

Upon arrival in the operating room, standard monitoring was initiated. A nociception monitor [the Analgesia Nociception Index (ANI), Mdoloris Medical Systems, Loos, France] was used for all patients. Anaesthesia was induced with propofol (2–4 mg kg^−1^) and sufentanil (0.2–0.3 μg kg^−1^). The use of a neuromuscular blocking agent for tracheal intubation was left to the discretion of the attending anaesthetist, in accordance with institutional practice. The lungs were mechanically ventilated, with a target end-tidal carbon dioxide pressure of between 35 and 40 mmHg. Anaesthesia was maintained using sevoflurane in a 40/60 oxygen/air mixture. Intraoperative analgesia was considered satisfactory when the ANI was >60,^13^ otherwise, 5–10 μg of sufentanil were re-injected. As per our routine institutional procedures for multimodal analgesia, all patients received i.v. dexamethasone (8 mg) at the beginning of surgery, and paracetamol (1 g), ketoprofen (100 mg) and nefopam (20 mg) at the end of surgery. Patients were extubated in the operating room and transferred to the postanaesthesia care unit (PACU). Patients received i.v. morphine in the PACU and oral paracetamol, tramadol and/or (if required) morphine on the ward if the patient's score on a numerical pain scale (NPS, from 0 to 10) was >3.

### Diaphragm movement and respiratory function

Diaphragmatic excursion was assessed by a blinded investigator before and 30 min after the ISB completion, using a low intercostal approach and a low-frequency (1.5–5 MHz) curvilinear ultrasound transducer (GE C1–5 RS) connected to the LOGIC P9.^14^ The low intercostal approach was preferred to the subcostal approach; in our experience, it often provides better images in obese patients and is better tolerated. The patient was examined in a 30° semi-recumbent position, and the hemidiaphragm was identified as a hyperechoic line with breathing-related movements along the lateral thoracic chest wall on the mid-axillary line. Hemidiaphragmatic excursion was measured using real-time M-mode ultrasonography during a deep, quiet breath from the resting expiratory position. The measurement was performed twice, and the highest value of each variable was kept for analysis. All measurements were repeated on the contralateral hemidiaphragm.

Respiratory function was assessed before and 30 min after the ISB, with the patient in a semi-recumbent position. A portable spirometry system (SpiroUSB, CareFusion, San Diego, CA, USA) was attached to the patient. After the patient had been told how to perform the test, forced vital capacity (FVC), FEV1, and peak expiratory flow (PEF) were recorded using dedicated software (Spirometry PC Software, CareFusion, San Diego, CA). The test was performed twice, and the highest value of each variable was kept for analysis.

### Outcomes

The study aimed to compare the experimental and control groups with respect to ipsilateral hemidiaphragmatic excursion after ISB. Excursion was measured at baseline and 30 min after ISB and expressed as the percentage change from baseline. Hemidiaphragmatic dysfunction was defined *a priori* as a >25% decrease in excursion from baseline at 30 min and was the primary outcome (incidence of dysfunction). Dysfunction was further categorised as hemidiaphragmatic paresis (25–75% decrease) or hemidiaphragmatic paralysis (>75% decrease); the incidence of hemidiaphragmatic paresis and paralysis were considered as secondary outcomes.

The study's secondary objectives were to compare the experimental and control groups with regard to (i) the respiratory function 30 min after ISB; the related outcomes measures were the changes in FVC, FEV1, and PEF 30 min after ISB. The incidence of respiratory dysfunction (defined as a decrease in FCV of more than 25%, compared with baseline) was also analysed; (ii) the changes in the course of the contralateral hemidiaphragm 30 min after ISB; (iii) intraoperative analgesia (intraoperative sufentanil consumption and the value of the ANI), and postoperative analgesia: the main outcome measure in this respect was the duration of effective analgesia (i.e. the time interval between completion of ISB and the first postoperative NPS score >3. Surgery was frequently performed on an outpatient basis, all patients were thus asked to note the first time at which they would estimate their pain to be at an NPS score >3. An unplanned analysis also considered the NPS scores recorded in the wards by the nurses to analyse this end-point. Morphine consumption in the PACU and the number of patients who required morphine administration postoperatively were recorded. The occurrence of dyspnoea or pulse oximetry <90% within 30 min after performing the ISB or in the PACU was also recorded. All data recorded after discharge to home were collected by telephone 24 h after surgery by an investigator who was blinded to the group allocation. Lastly, patient satisfaction with anaesthesia and analgesia was evaluated on a numerical rating scale (ranging from 0, unsatisfied, to 10, highly satisfied) 24 h after surgery.

### Statistical analysis

Based on the literature data and preliminary observations, the incidence of hemidiaphragmatic dysfunction (paresis or paralysis) following ISB with a low-volume of ropivacaine 0.5% was estimated to be at least 50%. An absolute risk reduction of 35% (corresponding to a target incidence of 15% with ropivacaine 0.1%) was deemed to be clinically meaningful. A total sample size of 52 patients (26 per group) was calculated as being able to detect this difference with a power of 80% and a significance level of 0.05 in a two-sided test. To account for potential drop-out, 30 patients were enrolled in each group.

Quantitative variables are reported as median (25th to 75th percentile) and categorical variables are reported as the frequency (percentage). All analyses were done in the intention-to-treat population, defined as all randomised participants analysed according to their allocated intervention.

For the primary endpoint, the incidence of hemidiaphragmatic dysfunction was compared between groups using the *χ*^2^ test, and the treatment effect was expressed as relative risk (RR) with 95% confidence interval (CI) for the experimental group vs. the control group. Secondary binary outcomes were compared between groups using the *χ*^2^ test (or Fisher's exact test when expected cell counts were <5), and treatment effects were similarly expressed as RRs.

Secondary quantitative outcomes were compared between groups using the Mann–Whitney *U* test. Effect sizes were reported as generalised odds ratios (GenORs). GenOR was defined as the odds that a randomly selected participant in the experimental group had a higher outcome value than a randomly selected participant in the control group.

As *post hoc* sensitivity analyses requested during peer review, adjusted between-group RRs for the primary and secondary binary outcomes were additionally estimated using modified Poisson regression models with a log link and robust (sandwich) standard errors, including the corresponding baseline value as a covariate. For paired continuous outcomes measured at baseline and postintervention, between-group differences in absolute change from baseline were estimated using linear regression including treatment group and the baseline value; results were reported as baseline-adjusted mean differences with 95% CIs.

Duration of effective analgesia (time to first NPS score >3) was analysed using time-to-event methods. Kaplan–Meier curves were used to describe the distribution of times in each group and compared using the log-rank test. Treatment effects were estimated using a Cox proportional hazards model, reported as a hazard ratio (HR) with 95% CI for the experimental vs. control group; the proportional hazards assumption was assessed using Schoenfeld residuals. In addition, restricted mean survival time (RMST) up to 24 h was estimated and the between-group RMST difference with 95% CI was reported. As the endpoint was administratively bounded by design, participants who remained pain-free at 24 h were censored at 24 h.

All statistical tests were two-sided and *P* < 0.05 was considered statistically significant. Because of the potential for type 1 error due to multiple comparisons, findings for analyses of secondary outcomes should be considered as exploratory. Analyses were performed using SAS software, version 9.4 (SAS Institute Inc., Cary, NC, USA).

## Results

A total of 60 patients (30 in each group) were recruited (Fig. 1). Surgery was performed on an outpatient basis in 13/30 and 14/30 patients in the experimental and control groups, respectively (*P* = 0.795). Ultrasound identification of structures of interest for ISB was good in all but one of the patients in the control group. In two patients (one in each group), arthroscopy was converted to open surgery, and in another (control group) patient, severe but brief hypotension (subsequently attributed to anaphylaxis) occurred at anaesthetic induction but did not prevent surgery. These three patients were not excluded from the main analysis as the study design specified an intention-to-treat analysis and the primary outcome was assessed prior to surgery. One patient received a second ISB in the PACU while his NPS score was 0 and was therefore censored at the time of the repeat block in the postoperative analgesia analyses. The two groups were well balanced with regard to patient characteristics, diaphragmatic movements, or ventilatory function at baseline, except for contralateral diaphragmatic excursion (Table 1).

**Table 1:** Patient characteristics and respiratory function at baseline

	Experimental group (ropivacaine 0.1%), *n* = 30	Control group (ropivacaine 0.5%), *n* = 30
Age, years	55 [46 to 56]	55 [37 to 61]
Sex (male)	17/30 (57%)	18/30 (60%)
Body mass index, kg m^−2^	27.5 [24.0 to 32.1]	26.9 [24.4 to 31.3]
ASA status		
1	11/30 (37%)	11/30 (37%)
2	18/30 (60%)	18/30 (60%)
3	1/30 (3%)	1/30 (3%)
Hypertension	8/30 (27%)	10/30 (33%)
Respiratory disease (non-severe)	0/30 (0%)	2/30 (7%)
Diaphragmatic excursion (homolateral), cm	2.2 [2.0 to 3.0]	2.3 (2.0 to 2.6)
Diaphragmatic excursion (contralateral), cm	2.0 [1.9 to 2.3]	2.3 (2.0 to 3.0)
Forced vital capacity, l	2.9 [2.2 to 4.0] (*n* = 29)	3.3 [2.4 to 4.4] (*n* = 29)
Forced expiratory volume in 1 s, l	2.6 [1.8 to 3.4] (*n* = 29)	2.8 [2.2 to 3.5] (*n* = 29)
Peak expiratory flow, l s^−1a^	5.3 [4.0 to 7.7] (*n* = 29)	6.0 [4.7 to 7.5] (*n* = 29)

Values are *n*/total *n* (%) or median [25th to 75th percentiles]; for quantitative variables the number of available cases are reported in bracket (*n*) in case of missing data.

ASA, American Society of Anesthesiologists.

The incidence and amplitude of hemidiaphragmatic dysfunction 30 min after ISB were significantly lower in the experimental group than in the control group (Table 2). For the primary outcome (a decrease in diaphragm excursion >25% from baseline), the incidence was 7 out of 30 (23%) in the experimental group and 29 of 30 (97%) in the control group (*P* < 0.001), corresponding to a relative risk of 0.24 (95% CI 0.13 to 0.46). Similar effect estimates were observed in the baseline-adjusted sensitivity analyses (Table 2).

**Table 2:** Changes in diaphragmatic excursion 30 min after the interscalene block

			Unadjusted analysis		Baseline-adjusted analysis	
	Experimental group (ropivacaine 0.1%), *n* = 30	Control group (ropivacaine 0.5%), *n* = 30	Effect size (95%CI)	*P*	Effect size (95%CI)	*P*
Any hemidiaphragmatic dysfunction (>25% decrease vs. baseline)	7/30 (23%)	29/30 (97%)	0.24 [0.13 to 0.46]^b^	<0.001	0.24 [0.12 to 0.48]^b^	<0.001
Hemidiaphragmatic paralysis (>75% decrease vs. baseline)	1/30 (3%)	25/30 (83%)	0.04 [0.01 to 0.28]^b^	<0.001	0.04 [0.01 to 0.29]^b^	0.002
Hemidiaphragmatic paresis (25% to 75% decrease vs. baseline)	6/30 (20%)	4/30 (13%)	1.50 [0.47 to 4.78]^b^	0.49	1.54 [0.46 to 5.19]^b^	0.49
Hemidiaphragmatic excursion						
Percentage relative change from baseline	-22 [-24 to -18]	-100 [100 to -100]	16.8 [5.2 to 54.3]^c^	<0.001	NA	NA
Absolute change from baseline (cm)	-0.6 [-0.8 to -0.4]^a^	-2.2 [-2.4 to -2.0]^a^	NA	NA	1.6 [1.3 to 1.9]^d^	<0.001
Contralateral hemidiaphragmatic						
Percentage relative change from baseline	+26 [+17 to +40]	+30 [+20 to +43]	1.21 [0.67 to 2.19]^c^	0.55	NA	NA
Absolute change from baseline (cm)	0.6 [0.4 to 0.8]^a^	0.8 [0.6 to 1.0]^a^	NA	NA	-0.2 [-0.5 to 0.1]^d^	0.13

Values are *n*/total *n* (%) or median [25th to 75th percentiles] unless otherwise is indicated.

a Baseline adjusted mean value (95% CI). Effects sizes were assessed using three different measures.

b The relative-risk for binary outcomes variables.

c The generalized odds-ratio for quantitative outcomes and

d Baseline-adjusted mean differences.

CI, confidence interval; NA, not applicable.

Spirometry was less impaired in the experimental group than in the control group in both unadjusted and baseline-adjusted analyses (Table 3). No dyspnoea was reported at any time. In the PACU, pulse oximetry values <90% occurred in two patients in the experimental group and three patients in the control group; all episodes were immediately corrected with supplemental oxygen (2–4 l min^−1^ for 15–30 min). No additional respiratory interventions were required.

**Table 3:** Respiratory outcomes (pre- vs. post-ISB)

			Unadjusted analysis		Baseline-adjusted analysis	
	Experimental group (ropivacaine 0.1%), *n* = 30	Control group (ropivacaine 0.5%), *n* = 30	Effect size (95%CI)	*P*	Effect size (95%CI)	*P*
Decrease in the forced vital capacity						
Percentage relative change from baseline	−17 [−23 to −5] (*n* = 29)	−29 [−42 to −6] (*n* = 29)	0.52 [0.27 to 0.98]^b^	0.039	NA	NA
Absolute change from baseline (L)	−0.52 [−0.72 to −0.33]^a^ (*n* = 29)	−0.83 [−1.02 to −0.63]^a^ (*n* = 29)	NA	NA	0.30 [0.03 to 0.58]^d^	0.031
Decrease in the forced expiratory volume in 1 s						
Percentage relative change from baseline	−16 [−23 to −5] (*n* = 29)	−29 [−41 to −10] (*n* = 29)	0.53 [0.28 to 1.01]^b^	0.051	NA	NA
Absolute change from baseline (l)	−0.43 [−0.60 to −0.26]^a^ (*n* = 29)	−0.69 [−0.87 to −0.52]^a^ (*n* = 29)	NA	NA	0.26 [0.03 to 0.58]^d^	0.034
Decrease in the peak expiratory flow						
Percentage relative change from baseline	−11 [−22 to −3] (*n* = 29)	−22 [−37 to −3]	0.64 [0.34 to 1.18]^b^	0.15	NA	NA
Absolute change from baseline (l)	−0.81 [−1.32 to −0.29]^a^ (*n* = 29)	−1.25 [−1.76 to −0.74]^a^	NA	NA	0.44 [−0.30 to 1.18]^d^	0.227
Patients with a decrease in the forced vital capacity >25%	8/29 (28%)	18/29 (62%)	0.44 [0.23 to 0.86]^c^	0.009	0.39 [0.19 to 0.82]^c^	0.012

Values are *n*/total *n*(%) or median [25th to 75th percentiles] unless otherwise is indicated. For quantitative variables the number of available cases is reported in bracket (*n*) in case of missing data.

a Baseline adjusted mean value (95% CI). Effects sizes were assessed using three different measures.

b The generalized odds-ratio for quantitative outcomes.

c The relative-risk for binary outcomes variables.

d Baseline-adjusted mean differences.

CI, confidence interval; NA, not applicable.

The experimental and control groups did not differ with regard to median [IQR] intraoperative sufentanil consumption (0 μg [0 to 5] vs. 0 μg [0 to 0], respectively; *P* = 0.058) or the median ANI values (70 [62 to 74] vs. 71 [62 to 79], respectively; *P* = 0.74). There were also no differences between the groups regarding the use of neuromuscular blocking agents (15/30 vs. 10/30 patients; *P* = 0.29), median end-tidal concentration of sevoflurane (1.8% [1.6 to 2.1] vs. 1.9% [1.6 to 2.0]; *P* = 0.60) or median intraoperative mean arterial pressure (76 mmHg [68 to 85] vs. 73 mmHg [67 to 81]; *P* = 0.34).

In the PACU, an NPS score >3 was recorded in three patients in the control group and six in the experimental group, who therefore received i.v. morphine (2–10 mg in each group). The median morphine consumption in the PACU was 0 mg [0 to 0] in both groups; *P* = 0.19.

**FIGURE 2:**
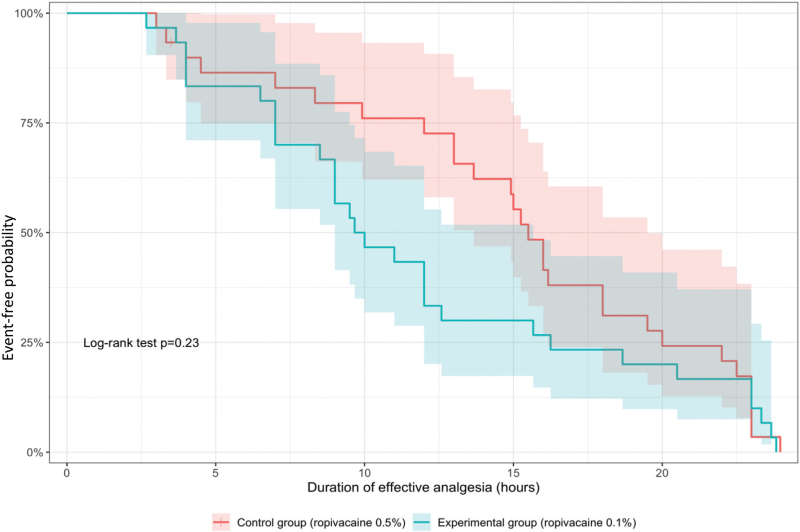
Kaplan–Meier estimates of the duration of effective analgesia in the experimental group (ropivacaine 0.1%) vs. the control group (ropivacaine 0.5%).

The median duration of effective analgesia was 9.8 h (95% CI, 9.0 to 15.7) in the experimental group and 15.5 h (95% CI, 13.7 to 19.5) in the control group. There was no evidence of a difference between groups according to the log-rank test (*P* = 0.23) (Fig. 2). Consistently, neither the Cox proportional hazards model nor the RMST estimation up to 24 h showed a statistically significant difference between the two groups (Table 4).

Each curve is shown with its corresponding 95% confidence interval (the shaded area). Event = numerical pain scale score >3.

**Table 4:** Duration of effective postoperative analgesia (defined as time to first numerical pain scale score >3)

	Experimental group (ropivacaine 0.1%)		Control group (ropivacaine 0.5%)						
	Median duration (h) (95% CI)	RMST (h) (95% CI)	Median duration (h) (95% CI)	RMST (h) (95% CI)	Log-rank *P*	Cox HR (95% CI)	Cox *P*	RMST mean difference (h) (95%CI)	RMST *P*
Patient-reported (no exclusion), *n* = 60	9.8 (9.0 to 15.7)	11.9 (9.6 to 14.3)	15.5 (13.7 to 19.5)	15.1 (12.8 to 17.4)	0.23	1.33 (0.79 to 2.24)	0.29	−3.2 (−6.4 to 0.2)	0.06
Nurse-assessed (no exclusion), *n* = 60	10.8 (6.5 to 20.5)	12.4 (9.5 to 15.3)	15.0 (10.0 to 18.0)	13.5 (11.1 to 15.8)	0.61	0.89 (0.53 to 1.50)	0.66	−1.0 (−4.8 to 2.7)	0.59
Patient-reported (with exclusion^a^), *n* = 57	10.0 (9.0 to 15.7)	12.1 (9.7 to 14.5)	15.2 (13.0 to 19.5)	14.7 (12.3 to 17.1)	0.63	1.09 (0.63 to 1.89)	0.75	−2.6 (−6.0 to 0.8)	0.13
Nurse-assessed (with exclusion^a^), *n* = 57	11.7 (6.5 to 20.5)	12.6 (9.6 to 15.6)	15.0 (10.0 to 18.0)	13.3 (10.9 to 15.7)	0.36	0.79 (0.45 to 1.37)	0.40	−0.7 (−4.5 to 3.1)	0.72

95% CI, 95% confidence interval; HR, Cox hazard ratio for pain onset (experimental vs. control); RMST, restricted mean survival time up to 24 h. See text for details on statistical analysis.

RMST mean difference = experimental − control.

a Exclusion of two patients whose arthroscopy was converted to open surgery (1 in each group) and one patient (control group) who experienced intraoperative anaphylaxis.

The analyses based on pain assessments by nurses during the ward stay found no differences between groups: 10.8 h (95% CI 6.5 to 20.5) in the experimental group and 15.0 h (95% CI 10.0 to 18.0) in the control group, as shown in Table 4.

Four patients in the experimental group required an oral dose of morphine in the ward, bringing the total number of patients who received morphine after surgery in the experimental group to 10 (compared with three in the control group, *P* = 0.028). All patients in the experimental and control groups were very satisfied (NPS score: 9 [9 to 10] vs. 10 [9 to 10], respectively; *P* = 0.96).

An additional sensitivity analysis, conducted in response to peer review, re-evaluated postoperative analgesic efficacy after excluding the two patients whose arthroscopy was converted to open surgery and the patient who experienced intraoperative anaphylaxis. Findings for the duration of effective analgesia were consistent with the primary analyses (Table 4); however, only 9/29 patients in the experimental group received morphine, compared with 3/28 in the control group (*P* = 0.06).

## Discussion

The results of this double-blind, controlled, randomised trial showed that the administration of a low volume of ropivacaine 0.1% during ISB reduces the incidences of hemidiaphragmatic dysfunction and lower the impact on respiratory function, compared with use of the same volume of ropivacaine 0.5%. The duration of effective analgesia was not significantly shorter with ropivacaine 0.1% than with ropivacaine 0.5%, but more patients required morphine during the postoperative period in the ropivacaine 0.1% group.

Several studies have investigated strategies to reduce the incidence of ISB-induced hemidiaphragmatic paralysis by modifying the type, concentration, volume, or injection site of local anaesthetics.^3,8–12,15^ Overall, lower volumes or concentrations were associated with reduced diaphragmatic and respiratory impairment, while postoperative analgesia was generally comparable between groups. Although low-volume or extrafascial techniques achieved a reasonable balance between diaphragm sparing and analgesia, reported paralysis rates still ranged from 20% to 40%. A recent study showed that reducing the extrafascial volume of ropivacaine 0.75% from 20 to 10 ml decreased the incidence of hemidiaphragmatic paralysis to 19%, at the expense of shorter analgesia duration.^16^ Notably, previous studies reported paralysis but not paresis. In our study, 7 ml of ropivacaine 0.5% in the control group resulted in hemidiaphragmatic paralysis in most patients, consistent with earlier reports. The rates of paralysis and paresis observed in the experimental group were comparable to those reported with so-called diaphragm-sparing blocks.^6,17^

The spirometry results in the ropivacaine 0.5% group are similar to those published in the literature, i.e. a post-ISB decrease in all variables studied. The lower degree of impairment in the experimental group is consistent with the ultrasound results. In the population as a whole, the alteration in FCV correlated with the amplitude of hemidiaphragmatic dysfunction (data not shown). The slight decreases in FCV, FEV1 and DEP observed in the experimental group were recorded for overweight patients: more than one in four of the patients had a BMI >30 kg m^−2^, and the highest value was 44 kg m^−2^) (Table 1). These results are reassuring with regard to the feasibility of ISB with a low volume and low concentration of local anaesthetic in patients with a low respiratory risk.

There was no significant difference in sufentanil consumption or intraoperative nociception (as estimated with the ANI) between the experimental and control groups. In fact, the dose of sufentanil was low in both groups. Postoperative analgesia duration was not different between the two groups, but the study was not powered to demonstrate noninferiority. The observed median difference of 4–5 h nonetheless raises questions about the risk–benefit balance of using low-volume, low-concentration ropivacaine for ISB. Morphine requirements in the PACU and wards were relatively low but tended to be higher in the experimental group. Patient satisfaction was high at the postoperative day 1 interview, which might suggest effective analgesia. However, as all patients received i.v. dexamethasone and multimodal analgesia, the observed effects cannot be attributed to the ISB alone.

The main limitation of our study concerns the assessment of intra- and postoperative analgesia. The usefulness of ANI for reducing intraoperative opioid administration remains controversial,^18^ but it has been consistently shown to early detect nociception. The absence of difference in intraoperative arterial pressure also suggests that the analgesia-nociception balance was not different between the two groups. The definition of an effective analgesia duration based on the first NPS score >3 has been used in other studies,^16^ but the use of a specific threshold remains subject to imprecision. Overall, only four patients in the experimental group required a single dose of morphine after discharge from the PACU, and satisfaction scores were high in both groups; however, as previously stated, the study was not powered to draw firm conclusions regarding postoperative analgesia. Secondly, diaphragmatic measurements and spirometry were performed only twice at each study time point, which may have reduced the likelihood of capturing maximal patient effort compared with studies in which three measurements were obtained and the highest value retained. However, since all measurements were performed under blinded conditions, it is unlikely that this potential limitation biased the results. Also, as with most previous studies of ISB in general and so-called ‘diaphragm-sparing’ blocks for shoulder surgery in particular, hemidiaphragmatic paralysis was not eliminated in our experimental group. As mentioned above, our results are reassuring for ISB in patients with a body mass index of up to 40 kg m^−2^. Patients with severe pulmonary disorders were not, however, included in our study, and no conclusions can be drawn for that patient population, which limits the clinical relevance of the study (in the same way as previous studies). Finally, ISB with low volume of a low concentration of anaesthetic (as tested in the present study) might not be appropriate for surgical anaesthesia.

In conclusion, ISB with 7 ml of ropivacaine 0.1% markedly reduces the risk of hemidiaphragmatic paralysis during arthroscopic shoulder surgery, when compared with the same volume of ropivacaine 0.5%. The lower dose only slightly impaired respiratory function and appeared to be associated with acceptable postoperative analgesia, but may have been less effective than the higher dose.

## Supplementary Material

Supplemental Digital Content
