# Supplementary figures and images for: The diaphragm-sparing effect of interscalene block with a low-volume of ropivacaine 0.1% vs. 0.5%: A double-blind, controlled, randomised trial
Source: Eur J Anaesthesiol Intensive Care. 2026 Apr 23;5(3):1-9. doi: 10.1097/EA9.0000000000000108 (PMC13232923; doi:10.1097/EA9.0000000000000108)

## Slide 1
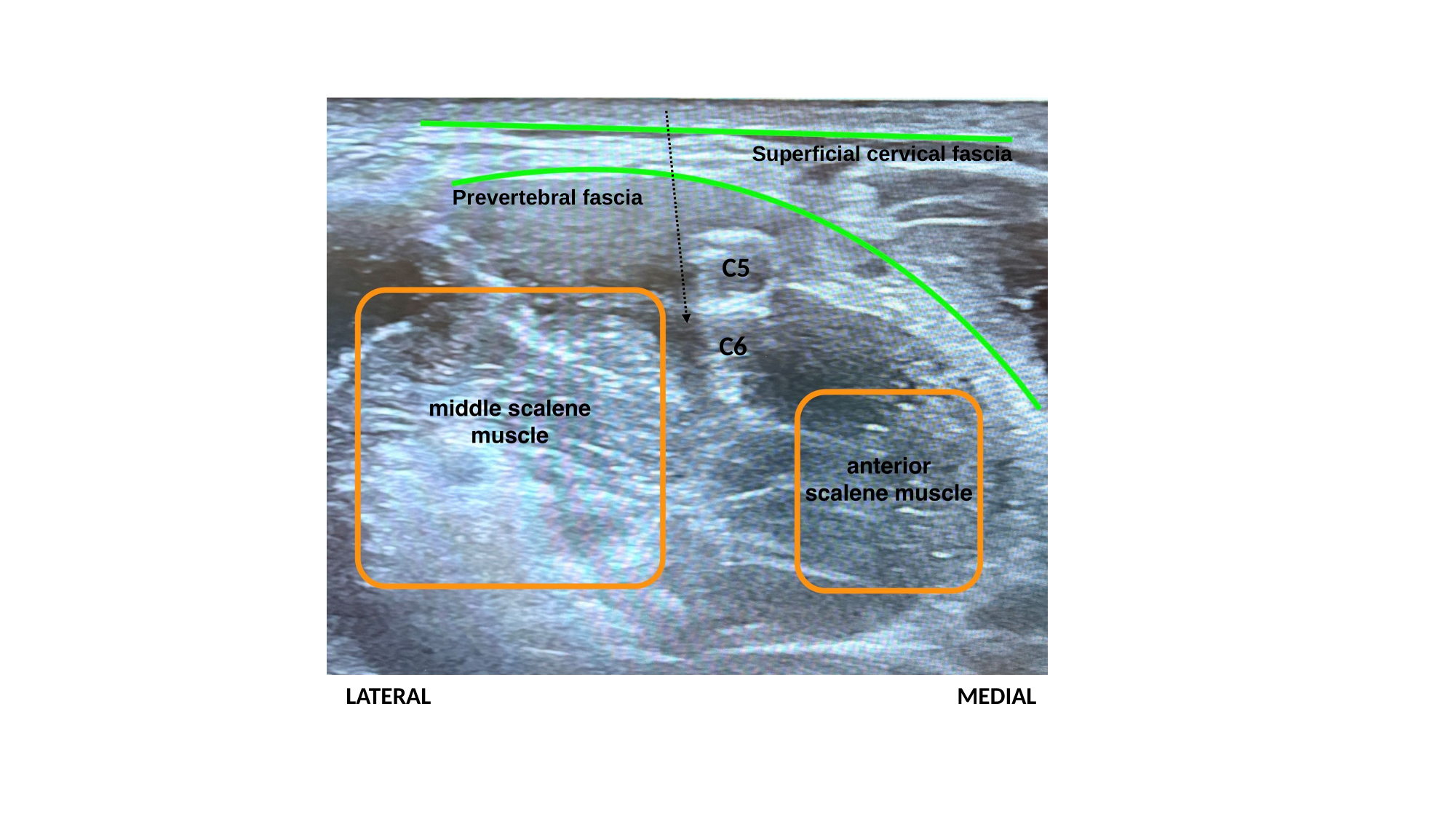

Superficial cervical fascia
Prevertebral fascia
C5
C6
LATERAL
MEDIAL

Supplement: Supplemental Digital Content [file ejaic-5-e0108-s001.pptx]
